# Cochlear Implants in Inner Ear Malformations—Considerations Regarding the Role of Imaging in Preoperative Evaluation

**DOI:** 10.3390/healthcare14131906

**Published:** 2026-07-01

**Authors:** Cristian Mircea Neagoș, Adriana Neagoș, Refka Dalia Najar, Răzvan Marian Melinte, Ana Fulga, Ileana Anca Sin

**Affiliations:** 1 Doctoral School of Medicine and Pharmacy, George Emil Palade University of Medicine, Pharmacy, Science and Technology, 540139 Târgu Mureș, Romania; neagos.cristian-mircea.23@stud.umfst.ro; 2 Department of Otorhinolaryngology, George Emil Palade University of Medicine, Pharmacy, Science and Technology, 540139 Târgu Mureș, Romania; 3 Department of Otorhinolaryngology, Emergency County Hospital of Târgu Mureș, 540136 Târgu Mureș, Romania; dalia.najar21@gmail.com; 4 Department of Orthopedy, Iuliu Hațieganu University of Medicine and Pharmacy, 400012 Cluj-Napoca, Romania; 5 Department of Otorhinolaryngology, Faculty of Medicine and Pharmacy, Dunarea de Jos University of Galati, 800010 Galati, Romania; 6 Department of Molecular Biology, George Emil Palade University of Medicine, Pharmacy, Science and Technology, 540139 Târgu Mureș, Romania

**Keywords:** cochlear implant, cochlea, computed tomography, vestibular aqueduct, incomplete partition, cochlear volume, cochlear malformations

## Abstract

**Background/Objectives**: Addressing cochlear malformations in the context of cochlear implantation is particularly important because the dimensions of the cochlea can affect intraoperative surgical decisions and options as well as the postoperative outcomes of each patient. High-resolution computed tomography (CT) is routinely used to assess the dimensions of the cochlea and the presence of any structural malformations. The purpose of this paper is to evaluate the dimensions of the cochlea and its malformations by using imaging measurements and correlating the data obtained with certain types of medical devices used. **Methods**: A retrospective observational study was conducted, through the systematic analysis of computed tomography images, on 130 cochlear implants between 2014 and 2024 at the ENT Clinic in Târgu Mureș, Romania, analyzing 103 patients. Data related to the anatomical features of the cochlea and their dimensions were evaluated, with an emphasis on the volume of the malformed and non-malformed cochleas. The collected data were statistically analyzed, evaluating the volume of the cochlea in relation to the gender and age of the patients, as well as the presence of cochlear malformations related to the analyzed side—the right and left ear. **Results**: The age groups analyzed were 58% between 0 and 7 years, 14% between 8 and 17 years, and 28% between 17 and 68 years, with the minimum age for implantation being 10 months and the maximum age being 68 years. Analyzing the presence of malformations by gender, it was found that enlarged cochlear aperture occurs at a rate of 14.89% in females and 17.02% in males, while an enlarged vestibular aqueduct occurs at a rate of 4.26% in females and 8.51% in males. Incomplete partition types I and II have a frequency of 10.64% in women and 14.89% in men. Incomplete partition type I is equally present in 10.64% of women and men. Cochlear hypoplasia occurs at a low frequency of 2.13% in women and is not observed in men in our analysis. **Conclusions**: In this study, we analyzed cochlear volume variables between malformed and normal cochleae. Cochlear dimensions (width and height) did not show statistically significant variations between the right and left ears, both in malformed and non-malformed cochleae. The cochlear volume is consistent across the groups studied and is not influenced by malformations or the demographic variables analyzed. Cochlear malformations, according to our assessments, do not present contraindications for a cochlear implant, which nevertheless demonstrates that a preliminary imaging assessment is extremely useful for determining the type of implant that can be used.

## 1. Introduction

Addressing cochlear malformations in the context of a cochlear implant is particularly important because cochlear dimensions can affect intraoperative surgical decisions and postoperative outcomes for each patient. To assess cochlear dimensions and the presence of structural malformations, high-resolution computed tomography (CT) is routinely used for bone, with sections of 0.5–0.6 mm. The sections used are sagittal sections to identify the path of the facial and vestibulocochlear nerves, as well as the internal auditory canal and coronal sections, with reconstruction possibilities [1]. Physiologically, the anatomy of the cochlea consists of two and a half turns, with the modiolar section being the most important for evaluating the internal architecture of the cochlea and for distinguishing between its normal and malformed appearance [2]. The literature shows that the modiolus is a square or pentagonal structure at the base turn of the cochlea or between the base turn and the middle part of the cochlea [3]. In the imaging evaluation of the inner ear, the interscalar septa can be highlighted as thicker parts located between the inner wall of the cochlea and the modiolus, which differentiate between two and a half turns and two and three-quarter turns of the spiral [2]. The cochlear aperture is the structure that contains the cochlear nerve and represents the bony passage centered at the base of the modiolus. Another important section is the one that passes through the round window, which shows the possibility of basal, middle, and apical rotation of the cochlea. Considering the elements described above, it can be stated that sagittal section imaging is an indispensable tool in preoperative computed tomography evaluation for assessing patients proposed for cochlear implantation (CI). Sometimes, CT can be used to identify potential etiologies of hearing loss, evaluate temporal bone anatomy, and plan surgical techniques for cochlear implantation [3].

Computed tomography (CT) enables detailed surgical planning by providing clear images of the ear’s bone structures. It identifies anatomical variants important for surgery, such as the facial nerve path and the distribution of mastoid air cells, ensuring a safer and more effective approach [4]. Computed tomography (CT) is an essential tool in detecting contraindications for cochlear implantation, such as cochlear aplasia and atresia of the cochlear nerve canal or cochlear opening. Assessment of the cochlear opening is necessary to identify abnormalities of the cochlear nerve, as the presence of cochlear stenosis may indicate cochlear nerve deficiencies, which can influence the decision to perform cochlear implantation [5]. CT can also be used to assess the size of the internal auditory canal (IAC) and the presence of IAC stenosis or atresia, which are significant for cochlear nerve deficiencies. In addition, it is possible to identify bony labyrinth anomalies, such as Paget’s disease and otosclerosis, which may influence cochlear implantation [6,7].

Preoperative evaluation should identify and assess factors that may increase the difficulty of the surgical procedure, such as mastoid sclerosis, sigmoid sinus position, high jugular bulb, aberrant carotid artery, and, especially, a dehiscent or aberrant facial nerve. The size of the round window and the thickness of the bone in the potential cochlear implant placement area should be considered, as these measurements may influence the choice of implant type and help prevent postoperative complications [5,8]. Imaging-identified malformations of the inner ear are a common cause of congenital hearing loss. Imaging that highlights defects of the modiolus, interscalar septum, and Mondini-type malformations describes the most common malformations of the cochlea. In this context, modern imaging techniques play an essential role [9]. Multiplanar assessment, together with 3D reconstructions, has diagnostic value, influencing the decision to implant and minimizing surgical risks. They allow anatomical details to be obtained in order to select the most suitable electrode and the optimal surgical technique. Postoperative audiological results are also closely related to anatomical variations, for example, in Apert syndrome, which is a genetic disorder associated with hearing loss.

In patients with another genetic syndrome called Charge syndrome, studies have shown the presence of a rudimentary posterior semicircular canal, supporting the idea that the inner ear malformations present in Charge syndrome contribute to its specific clinical profile. As a result, early identification of these malformations can be an important guide in choosing interventions such as cochlear implants or early hearing aids [10].

The embryology of the inner ear must be understood, as must the existence of malformations associated with embryological changes. Computed tomography and magnetic resonance imaging are the methods used to evaluate inner ear malformations. Computed tomography excludes possible associations with middle or outer ear malformations. Magnetic resonance imaging is preferred when evaluating the membranous labyrinth or anomalies of the acoustic–vestibular nerve in the internal auditory canal. Cochlear implants, as a method of auditory–verbal rehabilitation, are the preferred option for severe and profound sensorineural hearing loss associated with certain cochlear malformations. Beforehand, the patient undergoes imaging tests, including computed tomography and nuclear magnetic resonance imaging, to determine the type of cochlear malformation and obtain cochlear measurements, which are important in choosing the type of implant. Before proceeding with the surgical approach to cochlear implantation, it is important that the patient undergoes a thorough evaluation, including audiological tests and medical examinations, to determine eligibility and to assess the potential benefits of this intervention [11]. In addition to these aspects, cochlear implants are useful for improving communication skills and enhancing the quality of life for these patients [11]. The purpose of this study is to evaluate cochlear dimensions, assess cochlear malformations using imaging measurements, and correlate these measurements with certain types of implants or medical devices. The volumes of the malformed and non-malformed cochleae were compared between the right and left ears, and the results were reported by patients’ gender and age.

## 2. Materials and Methods

A retrospective observational study was conducted by systematically analyzing computed tomography images of patients before cochlear implant surgery. This study was conducted during the period 2014 to 2024 at the ENT Clinic in Târgu Mureș, Romania. In total, 103 ears were analyzed.

The purpose of this paper is to analyze the dimensions of the cochlea using imaging data by measuring its width and length and calculating its volume. Inner ear malformations were also analyzed according to the Sennaroglu classification [1]. The evaluation of computed tomography images also aims to identify cochlear malformations. The aim is to understand the role of preoperative imaging in intraoperative decision-making and to identify the main cochlear malformations and correlate them with variations in cochlear volume and the type of implant used. The information analyzed included demographic data on patients’ age and gender. Measurements of the normal cochlea were taken, such as the width of the cochlea measured at its base and the length of the cochlea obtained by measuring from the base to the top of the cochlea. To measure the height of the cochlea, from the base of the modiolus to the helicotrema, we looked for and used the mid-modiolar section. When measuring the base of the cochlea, we identified the section in which the round window was visible, marking the beginning of the base. To calculate the cochlear volume, we assimilated the cochlea with the geometric shape of a cone, divided the width obtained by 2 to obtain the radius of the cone base, and then to calculate the cochlear volume, we used the formula V = (πR^2^h)/3, where V = cochlear volume, π = 3.14, R = radius of the cochlea base, and h = height of the cochlea. All measurements were expressed in millimeters. Similar measurements were performed on non-malformed cochleae according to the Sennaroglu classification. In patients with malformed cochleae, the modiolar section was predominantly used as a marker for the measurements. We evaluated the cochlear appearance without considering the unilateral or bilateral implant appearance. We relied only on the cochlear aspects. The comparison made between the right and left ear is explained by the fact that the two cochleae were selectively examined.

Statistical analysis software was used to analyze the collected data: Microsoft Office Excel and GraphPad Prism 10. We used statistical tests in GraphPad. The volume of the cochlea was compared between the right and left ears, both malformed and non-malformed. This study was approved by the Research Ethics Committee.

## 3. Results

**Figure 1:**
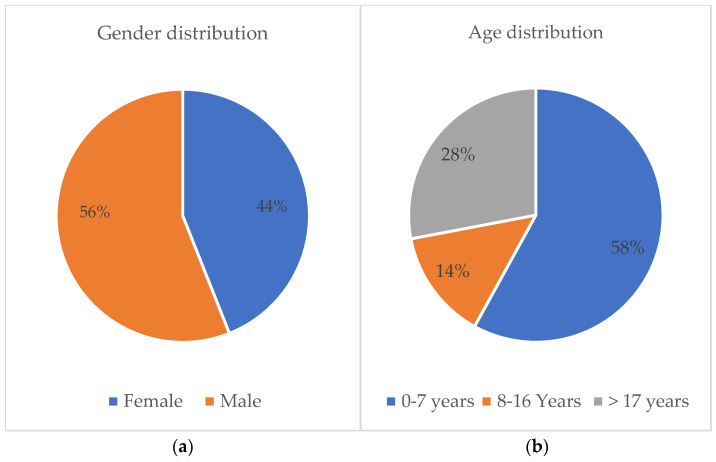
(**a**) Gender repartition of patients; (**b**) age repartition of patients.

Statistical analysis of the data based on imaging interpretation shows the following. Out of 103 cochleas analyzed, 44% (*n* = 45) were female, and 56% (*n* = 58) were male. The age groups analyzed were 58% (*n* = 60) between 0 and 7 years, 14% (*n* = 15) between 8 and 17 years, and 28% (*n* = 28) older than 17 years (Figure 1).

**Figure 2:**
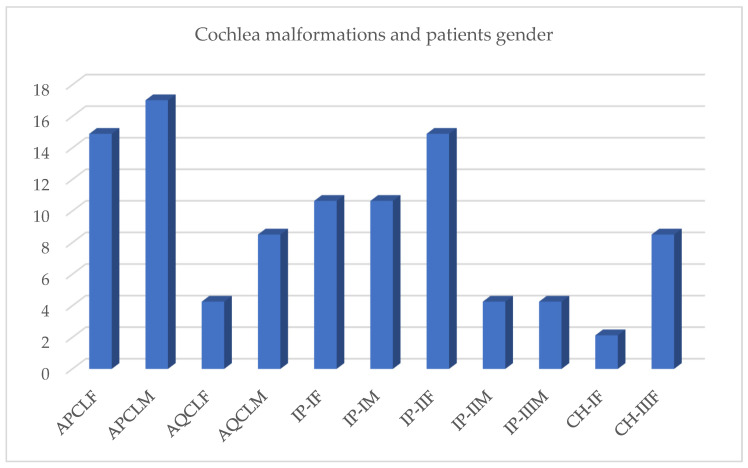
Distribution of cochlear malformations by gender, reported as percentages. APCLF—enlarged cochlear aperture in female patients; APCLM—enlarged cochlear aperture in male patients; AQCLF—enlarged cochlear aqueduct in female patients; AQCLM—enlarged cochlear aqueduct in male patients; IP-IF—type I incomplete partition in female patients; IP-IM—type I incomplete partition in male patients; IP-IIF—type II incomplete partition in female patients; IP-IIM—type II incomplete partition in male patients; IP-IIIM—type III incomplete partition in male patients; CH-IF—type I cochlear hypoplasia in female patients; CH-IIIF—type III cochlear hypoplasia in female patients.

**Figure 3:**
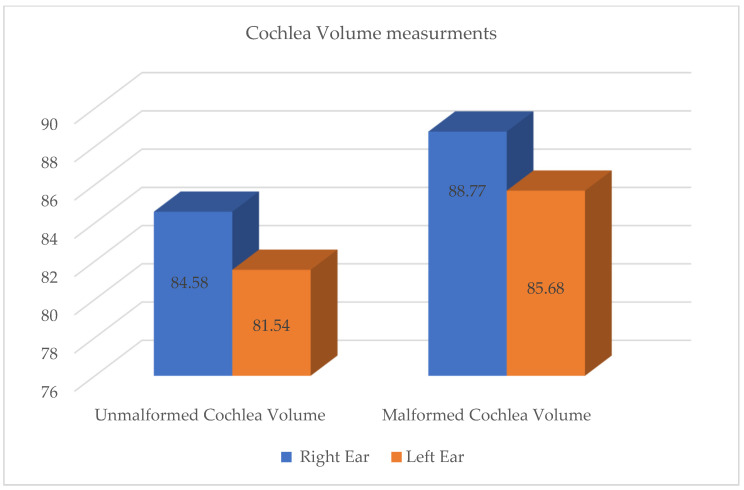
Cochlea volume measurements in malformed and non-malformed cochleae.

**Figure 4:**
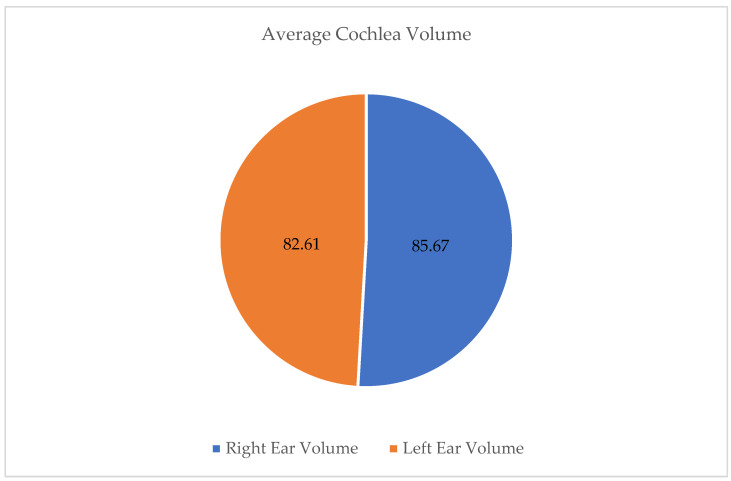
Comparison between average values of cochlear volumes in mm^3^.

1. Analyzing malformations by gender, it was found that enlarged cochlear aperture occurs in 14.89% of cases of females (*n* = 15) and in 17.02% (*n* = 17) of males, while enlarged vestibular aqueduct occurs in 4.26% of females (*n* = 4) compared to 8.51% (*n* = 6) of males. Incomplete partition types I and II have a frequency of 10.64% in males (*n* = 11) and 14.89% (*n* = 11) in females. Incomplete partition type I is equally present in 10.64% of females and males. Cochlear hypoplasia occurs at a low frequency of 2.13% (*n* = 2) in women and is not observed in men. Incomplete partition type II appears in 14.89% of female patients (*n* = 15) and 4.26% of males (*n* = 4). Cochlea hypoplasia is observed in females in 8.51% (*n* = 8) (Figure 2).

2. This study also included an analysis of the cochlear volume measured for four distinct groups: VCND (non-malformed cochlear volume of the right ear), VCNS (non-malformed cochlear volume of the left ear), VCMD (malformed cochlear volume of the right ear), and VCMS (malformed cochlear volume of the left ear). The data obtained can be described as follows: VCND has a mean value of 84.58 mm^3^ and a standard deviation of 14.92 mm^3^. VCNS has a mean value of 81.54 mm^3^ with a standard deviation of 15.48 mm^3^. Comparing cochlear volume between malformed and unmalformed cochleae separately in the right and left ears, it was observed that for the VCND and VCMD groups and the VCNS and VCND groups, respectively, there was an insignificant difference between the unmalformed and malformed cochleae in the right ears. The same statistical analysis, comparing VCNS and VCMS, showed an insignificant difference between the unmalformed and malformed cochleae in the left ears (Figure 3).

3. Comparing the volumes of the right and left cochleae, considering whether they are malformed or not, the following results were obtained: The average cochlear volume of the right ear was 85.67 mm^3^, with a standard deviation of 16.34 mm^3^. The same measurement on the left side showed an average cochlear volume of 82.61 mm^3^, with a standard deviation of 15.23 mm^3^ and a standard error of 1.523 mm^3^ (Figure 4).

## 4. Discussion

Based on the gender analysis of the statistical database, we can see an increased incidence of female patients (56%) and the predominant use of cochlear implants in the 0–7 age group, in line with the specialized literature. To analyze anatomical variations in cochlear malformations in relation to patients’ gender, we used several categories of cochlear malformations and compared their frequencies between women and men. Analyzing the data in the graphs and considering the previously evaluated clinical studies, we observe that the frequency of cochlear malformations differs between women and men. For example, the frequency of enlarged cochlear aperture in women (APCLF) is 14.89%, and in men (APCLB), it is 17.02%. A widened cochlear aqueduct in women (AQCLF) has a frequency of 4.26% and a frequency of 8.51% in men (AQCLB). Incomplete partition type I in women (IP-IF) and type II in women (IP-IIF) have an equal frequency of 10.64%, but incomplete partition type I in men (IP-IB) has a higher frequency (14.89%) compared to incomplete partition type II in men (IP-IIB) (10.64%). Type I cochlear hypoplasia in women (CH-IF) is present in a very small percentage of 2.13%, and type III cochlear hypoplasia in women (CH-IIIF) has a frequency of 4.26%. In men, type III cochlear hypoplasia (CH-IIIB) is present in 8.51% [2].

An analysis of cochlear volumes, comparing the right and left ears in both malformed and non-malformed cases, showed no statistically significant differences between these groups. The results show that the average volume of the non-malformed cochlea for the right ears is 84.58 mm^3^, and for the left ears, it is 81.54 mm^3^, with an insignificant difference of −3.041 mm^3^. Similarly, the average volume of the malformed cochlea for the right ears is 88.77 mm^3^, and for the left ears, it is 85.66 mm^3^, with a difference of −3.114 mm^3^, which is also insignificant.

These results suggest that there is no significant difference between the volume of the non-malformed and malformed cochlea between the right and left ears. The variability of cochlear volumes is relatively similar across all groups, indicating overall consistency in the measured data. Coefficients of variation ranging from 17.64% to 22.35% show stability in the measurements, and the relatively normal distributions of volumes suggest the absence of major deviations. The significance of these results, comparable to the literature studied, lies in the fact that cochlear malformations do not seem to significantly influence cochlear volume, and the differences observed between the right and left ears can be attributed to natural fluctuations and measurement errors, disregarding only cases of hypoplastic cochleae, which were not the focus of our study [3,5].

The interpretation of these results indicates that, anatomically, there are no significant variations in cochlear volume between the right and left ears, regardless of the presence or absence of malformations. This may suggest bilateral symmetry in cochlear development, which is important for understanding the norms of auditory structure development and may have clinical implications in the assessment and treatment of hearing disorders.

## 5. Conclusions

In this study, we investigated differences in cochlear volume between various groups and the influence of variables such as gender and malformations on cochlear dimensions. The results indicated no significant differences in the volumes of the non-malformed and malformed cochleae in both the right and left ears. Furthermore, no significant gender differences were observed among children, adolescents, and adults. Cochlear dimensions (width and height) did not show statistically significant variations between the right and left ears, both in malformed and non-malformed cochleae. These conclusions suggest that cochlear volume is consistent across the groups studied and is not influenced by malformations or the demographic variables analyzed.

Based on the data studied, no restrictions on cochlear implantation were identified for inner ear malformations.

## Data Availability

The data supporting the findings of this study include a retrospective observational clinical study based on CT images. The data are not publicly available because they include qualitative responses from a relatively small professional group, which may increase the risk of in-direct identification. An anonymized minimal dataset supporting the main findings can be made available to the editorial office for evaluation and to qualified researchers upon reasonable request to the corresponding author.

## Abbreviations

CT	Computed tomography
IAC	Internal auditory canal
APCLF	Enlarged cochlear aperture in female patients
APCLB	Enlarged cochlear aperture in male patients
AQCLF	Enlarged cochlear aqueduct in female patients
AQCLB	Enlarged cochlear aqueduct in male patients
IP-IF	Type I incomplete partition in female patients
IP-IB	Type I incomplete partition in male patients
IP-IIF	Type II incomplete partition in female patients
IP-IIB	Type II incomplete partition in male patients
IP-IIIB	Type III incomplete partition in male patients
CH-IF	Type I cochlear hypoplasia in female patients
CH-IIIF	Type III cochlear hypoplasia in female patients
VCND	Non-malformed cochlear volume of the right ear
VCNS	Non-malformed cochlear volume of the left ear
VCMD	Malformed cochlear volume of the right ear
VCMS	Malformed cochlear volume of the left ear
CH-I	Type I cochlear hypoplasia
CH-III	Type III cochlear hypoplasia
Type IC	Type IC—cochlear implant type
